# Non-Gaussian Liability Distribution for Depression in the General Population

**DOI:** 10.1177/10731911241275327

**Published:** 2024-09-09

**Authors:** Anna Talkkari, Tom H. Rosenström

**Affiliations:** 1 Department of Psychology, Faculty of Medicine, University of Helsinki, Finland

**Keywords:** PHQ-9, psychopathology risk distribution, Davidian-Curve Item Response Theory, complex quantitative trait, common mental disorders, semi-parametric estimation

## Abstract

Unlike depression sum scores, the underlying risk for depression is typically assumed to be normally distributed across the general population. To assess the true empirical shape of depression risk, we created a continuous-valued estimate of the latent depression density, using the Davidian-Curve Item Response Theory (DC-IRT) and the National Health and Nutrition Examination Survey (NHANES) cohorts from 2005 to 2018 (*n* = 36,244 on the Nine-item Patient Health Questionnaire; PHQ-9). We conducted simulations to investigate the performance of DC-IRT for large samples and realistic items. The method can recover complex latent-risk distributions even when they are not evident from sum scores. However, estimation accuracy for different sample sizes depends on the method of model selection. In addition to full-data analysis, random samples of a few thousand observations were drawn for analysis. The latent shape of depression was left-skewed and bimodal in both investigations, indicating that the latent-normality assumption does not hold for depression.

Although diagnostic manuals describe mental illnesses with binary categories, research on the underlying psychiatric constructs supports more continuous-valued representations (continuums or dimensions of symptom severity). The overwhelming majority of psychological variation appears dimensional rather than categorical (Haslam et al., 2020), including depression tendency (e.g., Liu, 2016; Ruscio & Ruscio, 2000). Attempts to find points of discontinuity between the non-depressed and depressed have received tentative support (Ruscio et al., 2007; Solomon et al., 2006), but the location of the cutoff point is ambiguous (Beach & Amir, 2006) and the conclusions can depend on the chosen mode of assessment (Ruscio et al., 2009). Data-driven approaches have not found support for symptom-based depression subtypes (van Loo et al., 2012) or biological subtypes (Beijers et al., 2019). Cluster analysis approaches have not given definitive answers on the latent structure of depression either, as symptom profiles differ from study to study (Bondar et al., 2020; Hybels et al., 2009; Schacht et al., 2014). A new approach to latent depression modeling is needed. This study will address this issue by estimating the latent density of depression in the general population from a semi–non-parametric (SNP) standpoint.

In the field of behavioral genetics, liability for common psychiatric disorders is characterized as a continuous dimension. Falconer (1965) introduced the concept of a liability distribution for estimating the risk of falling ill with common diseases when they are not inherited in a simple manner, that is, with one or two known alleles that form clear categories. Liability is the product of multiple genes and environmental factors and is thus best represented with a single, continuous-valued quantitative dimension. More recently, Plomin et al. (2009) suggested that the heritability of psychiatric constructs is the product of multiple genes of relatively small effect. They argue that the qualitative disorders of diagnostic manuals are mere extremes of quantitative and measurable dimensions. Subdiagnostic depression symptoms and clinical depression are thus considered representations of the same latent liability dimension. More notably, Falconer (1965) and Plomin et al. (2009) theorize that, in the general population, this latent liability distribution is normally distributed (a.k.a., Gaussian).

The normality of the latent construct is typically assumed for more technical reasons in psychological research, for example, when applying common methods like structural equation models. This assumption, however, has not been demonstrated to hold for depression. On the contrary, recent advances in latent depression modeling have suggested non-normal distributions for depression risk. In the clinically depressed, for example, Rosenström, Ritola, et al. (2023) recorded non-normal treatment responses to internet psychotherapies. Investigations of some parametric families of distributions have also rejected the normal model in the general population (Magnus & Liu, 2018). Tomitaka (2020) found that assuming latent non-normality helps explain previously recorded skewed depression questionnaire sum scores. These findings are not trivial as violations of distributional assumptions can introduce bias to the results, such as inflate measured treatment outcomes (Rosenström, Ritola, et al., 2023). Nevertheless, the tentative evidence for non-normality is not enough for accurate applications and raises the question: what is the shape of depression in the general population then?

The covert (modeled) latent liability pertains etiology and can have a continuous-valued distribution, such as the Gaussian, that does not resemble the overt, often skewed, measurements with categorical or binary questionnaire items or their sum scores (cf. illustrative first figures by Verhulst & Neale, 2021 or by Rosenström, Ritola, et al., 2023). Estimation of a continuous-valued model for latent depression involves methodological challenges, however. Despite the likely dimensional nature of psychiatric constructs, they are most often measured with ordinal-valued symptom questionnaires. Occasionally, the assumption of latent construct continuity is considered sufficient for researchers to apply methods designed for continuous variables to the ordinal items. This, however, is not recommendable as it can introduce bias to the results. The risk for bias is especially high for highly skewed observed data, such as zero-inflated responses in a symptom questionnaire (Verhulst & Neale, 2021). Data like these are commonly found in depression research across the world and across different questionnaires (Reise et al., 2021; Tomitaka et al., 2015, 2019a, 2019b, 2019c; Tomitaka, Kawasaki, Ide, Akutagawa, Ono, & Furukawa, 2018; Tomitaka, Kawasaki, Ide, Akutagawa, Yamada, et al., 2018) possibly because most people do not show clinically significant symptoms of depression. Thus, both the continuity and the distribution of the latent depression variable can differ vastly from the observed data. Inconsistencies between the distributional qualities of the latent and observed variables can result in inaccurate conclusions and are also challenging for latent-trait modeling. Fortunately, they can be addressed in psychometric research.

In this study, we are approaching these challenges from a relatively novel perspective, with the Davidian-Curve Item Response Theory ([DC-IRT], Davidian & Gallant, 1993; Woods, 2015; D. Zhang & Davidian, 2001). We will fit a DC-IRT model to ordinal depression questionnaire data to estimate the latent, continuous-valued, depression liability distribution in the general population, instead of assuming it. The DC-IRT is an SNP method that allows for the simultaneous estimation of the latent density of interest with the other IRT parameters. Instead of a standard normal distribution, as typically seen with IRT models, DC-IRT uses Davidian’s SNP distribution estimate (Davidian Curve [DC]) for the latent factor. According to Woods and Lin (2009), especially when the Hannan–Quinn (HQ) criterion (Hannan & Quinn, 1979) is used for model selection, the method’s ability to estimate a latent non-normal density is good, but it is also able to detect a true latent normal density when such a distribution underlies the data. However, these recommendations are based on simulations of relatively small-sample sizes of at most 1,000 simulated individuals (e.g., Woods, 2006, 2015; Woods and Lin, 2009), whereas we have observations on tens of thousands of individuals and highly skewed ordinal-valued items. To our knowledge, the modeling procedure has not been investigated in contexts like this before. To fill in these gaps, we will fit DC-IRT to both observed and simulated data.

Indications of true latent normality would support the current models of depression liability and common methodological choices of psychometric research. A skewed distribution would be an equally interesting finding. According to Lucke (2015) and Reise and Rodriguez (2016), a right-skewed latent distribution can be an indication of a unipolar latent construct. Only the higher scores on a unipolar dimension represent something significant, and lower scores simply indicate a lack of the trait. They suggest that scoring high on gambling addiction measures reflects high impulsivity and scoring low merely a lack of significant impulsivity, instead of high self-control, for example. When it comes to depression, a right-skewed distribution could have implications analogous to the impulsivity example, whereas the left tail of a left-skewed latent distribution could imply a population of high resilience against depression or of a very low or non-existent depression risk (relative to normal distribution). Magnus and Liu (2018), for example, argue for the existence of such a non-pathological class at the low end of the risk. Defining the distribution of depression in the general population would thus provide novel information on depression as a construct. It would aid future research by supporting causal inference (Rosenström, Czajkowski, et al., 2023) and by allowing more accurate methodological decisions and assumptions, and it may even pave the way for more accurate predictions of depression onset.

To summarize, in this study, we will answer the following questions: (a) how accurately DC-IRT can produce the true latent density from skewed ordinal data and (b) of what shape is the density of latent depression in the general population.

## Method

### Data

The sample (*n* = 40,496) consisted of seven cross-sectional National Health and Nutrition Examination Survey (NHANES) cohorts from the years 2005 to 2006 (*n* = 5,334), 2007 to 2008 (*n* = 5,995), 2009 to 2010 (*n* = 6,360), 2011 to 2012 (*n* = 5,615), 2013 to 2014 (*n* = 5,924), 2015 to 2016 (*n* = 5,735), and 2017 to 2018 (*n* = 5,533). The NHANES data sets consist of non-institutionalized civilian U.S. adults of 18 years and older. Each cohort is a new sample and does not include earlier participants. All NHANES data sets are available for public use, and further details of the NHANES protocol are available elsewhere (T.-C. Chen et al., 2018, 2020; Johnson et al., 2013). After removing missing values, a total of 36,244 observations remained for analysis. Sample weights were not used in our study, as the required methodology does not yet exist for DC-IRT, and we considered the samples representative enough for our purposes. Hereby, we report how we determined our sample size, all data exclusions, all manipulations, and all measures in the study.

### Measures

Depressive symptoms were measured with the Nine-item Patient Health Questionnaire (PHQ-9). The PHQ-9 is not only clinically useful (Kroenke et al., 2001) but has also proven a reliable and valid method for detecting subclinical depressive symptoms in the general population (Kocalevent et al., 2013; Martin et al., 2006). The PHQ-9 items correspond to the diagnostic criteria of major depressive disorder (MDD) listed in the *Diagnostic and Statistical Manual of Mental Disorders* (5th ed.; DSM-5; American Psychiatric Association, 2013). Therefore, PHQ-9 items should reflect the true depression distribution if such a thing exists for the most frequently used construct.

The nine items measured loss of pleasure, low mood, insomnia and hypersomnia, fatigue, changes in appetite, feelings of worthlessness or guilt, concentration difficulties, changes in psychomotor expression, and thoughts of self-harm or death, respectively. Respondents used a 4-point ordinal response scale (“Not at all,”“Several days,”“More than half the days,”“Nearly every day”) to express how often they had experienced each depressive symptom during the past 2 weeks. The questionnaire was administered as a computer-assisted self-report.

### Statistical Approach

#### Simulations

To test DC-IRT’s ability to identify the latent shape from ordinal data, we created three types of continuous-valued data to serve as the simulated latent trait: Gaussian, right-skewed, and bimodal. All data had a mean of 0 and variance of 1/√2. We simulated nine items to reflect the latent variable by adding normally distributed unique variance of 1/√2 to each in addition to the shared latent variable, so that each item had variance of 1. Finally, we created ordinal-valued data from these simulated data by placing ordinal thresholds on the right tail of the distribution, so that the ensuing category endorsement frequencies corresponded to relative frequency of the PHQ-9 item-category endorsements in our real data. Hence, the cutoff points (thresholds) between the four response categories were focused on the positive end of the latent trait. All latent values below 0 were consequently grouped into the lowest category, leaving it “zero-inflated” in appearance. The DC-IRT was fitted to these ordinal data, and the HQ-best models’ distribution estimation accuracy was compared to the true latent shape with the integrated squared error (ISE). ISE is defined as follows:

$$\int_{-\infty }^{\infty }{\left\{\hat{g}\left(\theta \right)-g\left(\theta \right)\right\}}^{2}d\theta ,$$

where θ is the latent factor or variable of interest, $\hat{g}$ is its estimated probability density function, and *g* its true probability density, which is known for the simulated data.

We evaluated estimation accuracy for multiple simulated sample sizes. These were 500, 1,000, 2,000, 3,000, 4,000, 5,000, 6,000, 7,000, 8,000, 9,000, 10,000, 11,000, and 12,000 simulated individuals. We investigated distribution of ISE estimates over 50 samples of each size for the skewed and bimodal simulated data and over 200 samples for the Gaussian data. More samples were needed for Gaussian data due to the lower signal-to-noise ratio for low ISE values. Although our primary study questions pertained ISE, for comprehensiveness, we report supplementary simulation results on estimation accuracy for usual IRT parameters, such as item discrimination and person’s estimated standing on the latent continuum (Chalmers, 2012).

#### DC-IRT

We estimated DC-IRT as suggested by Woods and Lin (2009) for each original NHANES cohort and simulated data set. We used the Marginal Maximum Likelihood estimation by Bock and Aitkin (1981) which relies on the Expectation–Maximization (EM) algorithm, with some adjustments for the estimation of the IRT parameters. The EM algorithm proceeds by sequentially alternating the “E step” and the “M step.” In the E step, the log-likelihood function of the entire data is estimated by calculating predicted response frequencies to each item, marginalizing (taking an *expectation*) over the levels of the unobserved latent variable. This step required the response probabilities, that is, the item response functions, and the probability distribution of the latent trait of interest. The parameters are treated as known in each E step. In the M step, the parameters are tuned to maximize the log-likelihood function and then passed back on to the next iteration of E step. The E and M steps alternated until convergence, that is, until the peak of the log-likelihood function was reached. The EM algorithm is typically used with a standard normal distribution as the latent density, but here we used the DC estimator instead. The item response function was a graded response model (Chalmers, 2012; Samejima, 1997), in a close analogy to the two-parameter logistic function, as in Woods and Lin (2009). Hence, response probabilities in each four-category ordinal item are connected to a latent trait through a discrimination parameter and three-item intercepts that are related to the severity thresholds—a standard IRT solution for modeling ordinal items.

The DC is a combination of a standard normal distribution density function φ and a squared polynomial $P_{k}^{2}$ of order *k*. Formally, this means that the (non-standardized) density of the latent trait θ is expressed as follows:

$$h\left(\theta \right)=P_{k}^{2}\left(\theta \right)\varphi \left(\theta \right)={\left\{\sum_{\lambda =0}^{k}m_{\lambda }\theta^{\lambda }\right\}}^{2}\varphi \left(\theta \right),$$

with the constraint $E[P_{k}^{2}\left(Z\right)]\;=\;1$ , where *Z* is a standard normal variate. An avid reader may wish to look at few illustrative example density curves from the Supplementary Figure S1. A DC requires only one tuning parameter, the order of the polynomial (*k*), which also corresponds to the number of parameters (polynomial weights *m*_λ_) in each contesting DC model. For example, *k* = 2 is a two-parameter model, *k* = 6 is a six-parameter model, and so on (note, DC-IRT has further item parameters). We fitted DC-IRT models up to the 10th-degree polynomial, as models more complicated than this are usually unnecessary and can lead to overfitting. The above-mentioned constraints for the squared polynomial imply that DC orders *k* = 0 and *k* = 1 always produce the normal model in DC-IRT (Woods & Lin, 2009), so we fitted models of orders *k* = 2 to 10. Even two-parameter DC models can express countless alterations of the latent density, and technically, any of the nine contesting models can also produce the Gaussian model, if that is what fits the data best. The HQ criterion was then used to choose the best model because Woods (2015, p. 77) noted that “The HQ criterion has worked well for model selection in Davidian’s work (Davidian & Gallant, 1993; D. Zhang & Davidian, 2001), and for DC-IRT (Woods & Lin, 2009). The same advice given for model selection for RC-IRT applies to model selection with DC-IRT”, which was “analysts should seriously consider the HQ-best model for interpretation” (Woods, 2015, p. 73).

The R programming language version 4.1.3 (2022-03-10) was used for statistical analysis (see Supplementary Material for relevant R code). DC-IRT was conducted with the mirt R package (Chalmers, 2012), using default settings. The mirt package computes HQ criterion as follows:

HQ=−2log(f(X|Θ))+2dim(Θ)log(log(n)),

where dim(Θ) is the number of free model parameters, *n* is the sample size, and f(X|Θ) is the likelihood of the parameters given the observed data *X*. A smaller HQ indicates a better fit. The penalty term 2dim(Θ)log(log(n)) is the lower bound for model-complexity penalty terms that ensure consistent model selection, but Hannan and Quinn (1979) used the penalty 2c×dim(Θ)log(log(n)) with *c* > 1. This ensures consistency, but little guidance exists for choosing *c*. Other commonly applied information criteria include Akaike’s (AIC) information criterion and Bayesian information criterion (BIC). AIC uses a smaller penalty, 2dim(Θ) , and is more efficient but not consistent in model selection. BIC uses a larger penalty, 2dim(Θ)log(n) , being the least efficient estimator, but it is consistent and favors simple models (J. Zhang et al., 2023). We compared HQ, AIC, and BIC model selection in supplementary simulation results.

With two of the NHANES data sets (2007 to 2008 and 2009 to 2010), the EM steps did not converge for the sixth and tenth DC orders’ models (i.e., for 4 of the 63 fits from 7 NHANES samples and 9 DC-IRT models per sample). The convergence threshold for the EM algorithm was lifted from 0.001 to 0.01 to achieve convergence for these models as well. This did not remarkably affect the other estimates, and the HQ-best model remained unaffected in both the cases. We present the results as standardized DC densities (having mean 0 and variance 1), that is, after having applied change of variables formula to the original (mirt-estimated) DC density, as described by X. Zhang et al. (2021; their Equation 13) (see Online Supplemental for examples of our computational procedures).

## Results

### Simulations

**Figure 1.**
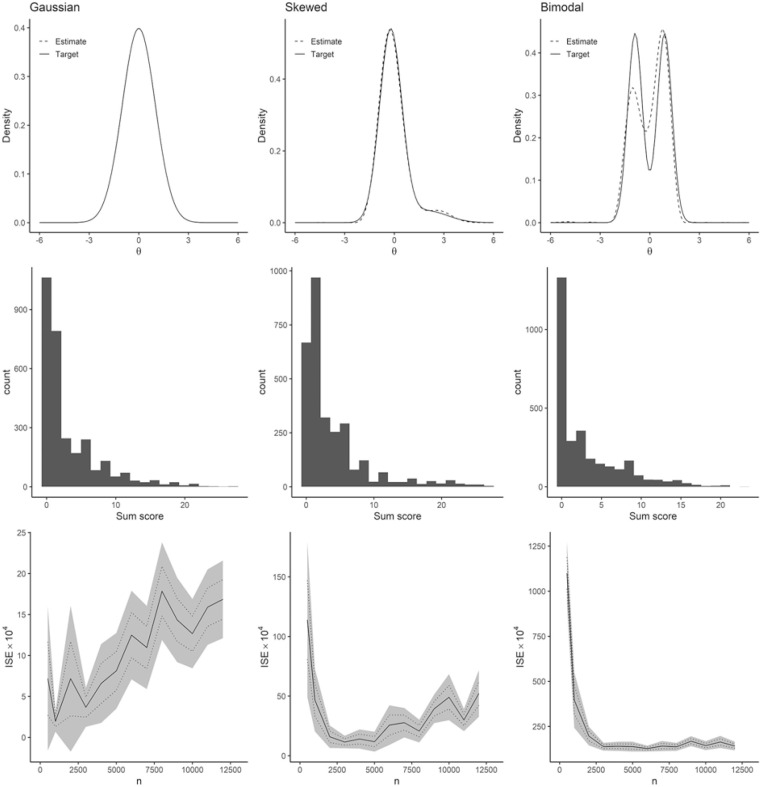
Simulation Results. Left column of panels for Gaussian (i.e., normal) data, middle column for skewed data, and right column for bimodal data. Top row shows the density of the data-generating simulation distribution (the target; solid line) and a single Davidian-Curve Item Response Theory (DC-IRT) model estimate from a model fitted to 3,000 simulated example observations (dashed line). Middle row shows histograms of sum scores, obtained for 3,000 simulated individuals from simulated ordinal responses to 9 items reflecting the indicated latent target distribution. Bottom row shows, for different sample sizes (x-axis), the average scaled integrated square error (ISE, y-axis, solid line) between the target latent distribution and its estimate. Dotted lines show the standard errors of the means, and the gray area shows the 95% confidence interval. Each sample size was simulated 50 times for the other distributions and 200 times for the Gaussian (due to the lower signal-to-noise ratio in the low ISE values). Note the different scales of y-axes of different panels.

Figure 1 summarizes results from the simulation test of our DC-IRT procedure. The first column corresponds to simulations of Gaussian (standard normal) distributed data, the second column to skewed data, and the third column to bimodal data. The first row of panels shows density functions of the data-generating distributions (solid lines) and one model fit to one simulated sample per distribution (dashed line; sample size 3,000 in each). Gaussian and skewed data were very well approximated by these DC-IRT models. The method could also detect the two modes in the bimodal simulated data, albeit the estimation accuracy appeared lower. This is quite striking as the two modes are in no way evident from the final simulated ordinal data to which the model was fitted (cf. middle row of Figure 1 for histograms of the sum scores of the simulated ordinal items). The mode detection performance was no coincidence either as 10 further simulations all led to similar densities (Supplementary Figure S2).

To comprehensively characterize the performance of DC-IRT, we plotted ISE against the sample size, across all the simulations (see bottom row of Figure 1). Several noteworthy patterns emerged. First, on average, the estimates were an order of magnitude more accurate for the Gaussian data compared to the skewed data, while also being an order of magnitude more accurate for the skewed data compared to the bimodal data (see scales of y-axes in Figure 1, bottom row). Second, for the non-Gaussian data, sample size 3,000 and above guaranteed comparably good estimation accuracies. Third, and most surprisingly, the estimation accuracy of DC-IRT for the latent density appeared to deteriorate for sample sizes 5,000 and above in the cases of Gaussian and skewed data. The deterioration at larger sample sizes appeared to be attributable to the HQ information criterion because it did not occur when not using it (Supplementary Figure S3; note, however, HQ criterion generally improved accuracy and reduced variance compared to using fixed DC order of 5, at least when *n* < 8,000).

Having pinpointed (the recommended) HQ criterion as a potentially problematic aspect of DC-IRT for large-sample sizes, we re-run the simulation by comparing HQ, AIC, and BIC model selection (Supplementary Figure S4). In contrast to HQ and AIC, model-selection performance did not deteriorate as the sample size grew when BIC was used. BIC was a consistently good model-selection criterion for large-sample sizes (*n*≥ 5,000), even though the previous research in smaller samples did not recommend it.

Regarding estimates for a (simulated) person’s standing on the latent trait, both DC-IRT and traditional IRT resulted in very similar mean-squared errors of estimation, expect for the skewed latent trait, where DC-IRT was superior to traditional IRT that modeled (an assumed) Gaussian latent trait (Supplementary Figure S5). DC-IRT also appeared superior for item parameters for the case of skewed latent distribution, but not for bimodal latent trait. HQ versus BIC model selection did not make much difference regarding person and item parameters.

### NHANES Data Sets

**Figure 2.**
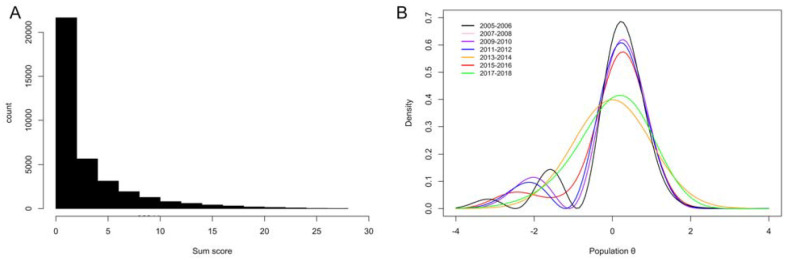
(A) PHQ-9 Sum Score Histogram for All NHANES Cohorts Combined and (B) Davidian-Curve Item Response Theory Estimates of Latent Densities for Each NHANES Cohort. *Note.* The 2007 to 2008 and 2009 to 2010 NHANES cohorts estimated latent densities strongly overlap, making only one of them visible in panel B.

For each NHANES data set, PHQ-9 sum score histograms were extremely skewed and more heavy-tailed than the standard normal distribution (Table 1). Figure 2A shows a histogram of the PHQ-9 sum scores for the entire data (i.e., for combined NHANES cohorts). The age range in each data set was 18 to 80+ [ages above 80 were top-coded] and the PHQ-9 sum score range 0 to 27, except for 2017 to 2018 NHANES, for which it was 0 to 25. Other sample demographics resembled the general population. Figure 2B presents estimates of the latent shape of depression for each NHANES cohort, based on the HQ-best DC-IRT models per cohort. Majority of the estimates were bimodal and left-skewed, but some also closer to a Gaussian shape. Most of the NHANES cohorts exceeded the optimal sample size suggested by the above simulation results to produce the best accuracy (when using HQ criterion).

**Table 1:** Observed-Data Properties

Survey years	*N* (female %)	PHQ-9 sum scores		
		Mean (SEM^a^)	Skew^b^	Kurtosis^c^
2005–2006	4,797 (51.8)	2.73 (0.05)	2.25	9.29
2007–2008	5,410 (50.4)	3.32 (0.06)	1.99	7.38
2009–2010	5,543 (50.2)	3.33 (0.06)	1.95	7.17
2011–2012	4,924 (49.6)	3.16 (0.06)	2.16	8.27
2013–2014	5,371 (51.9)	3.31 (0.06)	2.00	7.28
2015–2016	5,134 (51.1)	3.24 (0.06)	2.12	8.31
2017–2018	5,065 (51.1)	3.24 (0.06)	1.91	6.88
Total	36,244 (50.9)	3.20 (0.02)	2.05	7.76

a Standard error of the mean. ^b^Fisher’s moment coefficient of skewness. ^c^Pearson’s measure of (excess) kurtosis.

**Figure 3.**
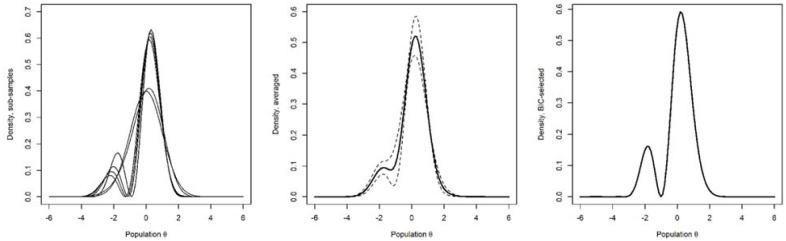
Davidian-Curve Item Response Theory Estimates of Latent Densities for 12 Non-Overlapping Random Subsamples of Size 3,000 From All the NHANES Participants and Over the Entire Data (n = 36,244). Left panel plots all the estimated subsample densities separately, some of which strongly overlap. Middle panel plots the pointwise average of these curves across the y-axis (solid line) and its 95% Wald confidence interval (between the dashed lines). Right panel shows an estimate over the entire data using the Bayesian information criterion–based model selection, which performed well also for large-sample sizes.

Motivated by our simulation findings (Figure 1) and to gain the best overall accuracy, we combined all the NHANES data sets and drew multiple random samples (without replacement) of size 3,000 from these data. Such samples avoid the increased HQ-related bias or variance of the estimator suggested by our simulations and balance out possible cohort effects. We then estimated DC-IRT in each of these subsamples. Figure 3 plots the latent-density estimates for all the 12 subsamples (left panel), and importantly, the (pointwise) average of these estimates (middle panel). The latter averaged estimate over the 12 subsamples of 3,000 individuals can be considered as our best HQ-based approximation for the true latent distribution of depression, if such a thing exists over time. In the simulations, BIC was the best model-selection criterion for large samples. Applied to the full real data (*n* = 36,244), it replicated the bimodal-density finding (Figure 3, right panel).

When the 12 subsample latent densities were used to derive skewness and kurtosis through numeric integration, the average skewness for the latent densities across the subsamples was −0.689 (95% Wald confidence interval [CI] = [−1.008, −0.369]) and average excess kurtosis was 0.801 (95% Wald CI = [0.345, 1.256]). Hence, the latent-trait distribution differs from a Gaussian much less than the PHQ-9 sum score (cf. Table 1) but nevertheless differs. It is skewed to the opposite direction compared to the sum score of the ordinal items. The numbers were similar for the BIC-based full-data model (skewness = −0.857, ex. kurtosis = 0.602).

Table 2 shows the PHQ-9 item discrimination parameters, severity intercepts, and factor loadings for the BIC-based full-data model. Implied factor loading estimates are compared to those from traditional IRT in the two rightmost columns. The generalized-severity column presents single location indices based on the item response functions of each item as defined by Ali et al. (2015, Equation 30). These general severity indices were included for ease of interpretation, but they and the factor loadings are not model parameters as such. Items measuring low mood, feelings of worthlessness or guilt, and suicidal ideation had the largest discrimination parameters, whereas items measuring insomnia and hypersomnia, changes in appetite, and fatigue had the lowest discriminative ability. Coincidingly, the severity index for suicidal ideation was the highest, whereas for fatigue, it was the lowest. These findings align with clinical practice, where suicidal ideation carries special weight when the PHQ-9 is used as diagnostic aid (Kroenke et al., 2001). For each of the PHQ-9 items, the factor loadings (discriminability) were stronger with DC-IRT as compared to traditional IRT that (wrongly) assumes a Gaussian latent depression density.

**Table 2:** Davidian-Curve Item Response Theory Item Parameters and Factor Loadings for All the NHANES Participants (*n* = 36,244) with Bayesian Information Criterion–Based Model Selection

PHQ-9 item	Discrimination (*a*)	Item intercepts			Generalized severity (*LI_IRF_*)^a^	Factor loadings	
		*d*_1_	*d*_2_	*d*_3_		DC-IRT	Traditional IRT
1. Loss of pleasure	3.13	−2.43	−4.41	−5.59	1.34	0.88	0.80
2. Low mood	4.32	−3.17	−5.81	−7.20	1.28	0.93	0.87
3. In-/hypersomnia	2.21	−1.02	−2.69	−3.55	1.11	0.79	0.70
4. Fatigue	2.66	−0.40	−2.90	−3.96	0.96	0.84	0.76
5. Changes in appetite	2.45	−2.04	−3.66	−4.63	1.42	0.82	0.73
6. Worthlessness/guilt	3.77	−3.63	−5.69	−6.84	1.44	0.91	0.84
7. Concentration problems	2.99	−3.07	−4.75	−5.70	1.52	0.87	0.78
8. Psychomotor problems	2.91	−3.77	−5.27	−6.27	1.76	0.86	0.78
9. Self-harm/death thoughts	3.23	−5.69	−7.20	−8.08	2.17	0.89	0.81

Note: The mirt package transforms a discrimination parameter *a* to a factor loading by taking $a/\sqrt{D^{2}+a^{2}}$ , where *D* is the constant 1.702 that aligns Logistic function with the cumulative normal distribution function. Factor loadings from traditional IRT are included for comparison.

a Single location index based on item response functions as described by Ali et al. (2015, Equation 30). *LI_IRF_* equals to the latent score that leads to the expected item score of *m*/2, where *m* is the highest item category.

## Discussion

We fitted the DC-IRT to both simulated and observed data to first understand the context-specific properties of the method and to then maximally accurately estimate the density function of latent depression in the general population. The simulations proved that the method finds complicated latent shapes even when the latent density is not at all evident from the ordinal data. The previously recommended HQ-based estimates were most accurate at sample sizes of ~3,000. Subsamples of this optimal size were drawn from the observed data for further analysis. Four of the 12 samples produced unimodal density estimates, whereas seven resulted in bimodal densities. On average, the latent shape of depression was non-Gaussian (not normally distributed) and bimodal. This shape was replicated in a BIC-based estimate on the full data (*n* = 36,244), and our simulations found BIC to be more accurate in large samples compared to the recommended HQ criterion.

According to the simulations, the DC-IRT can find latent distributions that are normal, skewed, and even bimodal from skewed ordinal item data. That is, the model can separate observed skewness related to item correlations due to a (skewed) latent factor from skewness related to mere item-specific measurement properties (i.e., ordinal-category thresholds). The ability to detect bimodal distributions was especially remarkable as the simulated ordinal data showed no visual indication of bimodality. Even when the first mode completely resided below the ordinal-category thresholds, the method was able to find two modes, indicating that DC-IRT is not bounded by the limitations of the measurement tool used (e.g., by floor effects). However, the estimator systematically underestimated the height of the first mode (Figure 1, estimate vs. target in the upper-right panel; see also Supplementary Figure S2). This statistical bias partially explains why the inaccuracy (i.e., ISE) values were significantly higher for the bimodal case. Estimate accuracy, as measured by inverse of the ISE between true and estimated shapes, peaked at around 3,000 observations for the normal and skewed data. Increasing sample size did not improve the results further, and in some cases, even made them less accurate.

We attribute the undesirable large-sample behavior of the DC-IRT estimator to the HQ information criterion it uses, without which the large-sample deterioration did not happen (Supplementary Figures S3 and S4). Unsurprisingly, small-sample performance was better when using the criterion. Previous studies have focused on relatively small samples (thousand or less) and, therefore, probably have entirely missed the large-sample problems that we found (see, e.g., Woods & Lin, 2009; X. Zhang et al., 2021). Despite the good overall performance, there is need for further methodological developments that reduce the method’s statistical bias and improve its large-sample behavior, if possible. In general, the best choice for an information criterion is a context-dependent and non-trivial problem, where potentially misleading folklore exists in the literature (J. Zhang, Yang, & Ding, 2023). Our supplementary simulation results indicated that BIC enjoyed good properties in large-sample sizes, whereas the HQ criterion may be preferable in samples of 3,000 observations and less (Supplementary Figure S4). BIC is known to be comparatively conservative regarding model complexity (Dziak et al., 2020), which may explain its success in large samples.

Our Figure 3 is the first SNP large-sample estimate of latent depression in the general population, comprising a total of 36,000 observations (36,244 for the BIC-based estimate; right panel). The estimate’s left-skewed shape might coincide with the “zero-inflation” of depression symptoms found in non-clinical samples (Magnus & Liu, 2022). Importantly, the discontinuity or skew is not where the current clinical models of depression would predict, that is, around the high values of latent depression. The transition to illness is frequently thought to occur when diagnostic criteria for severe enough symptom presence and frequency are met. However, our empirically estimated latent density does not suggest latent classes at the severe end of its domain. Our results suggest that the very low values of latent depression are somehow distinct instead of the high values that arguably separate the clinically depressed from the rest. This is an interesting finding that calls for further investigation.

Although we cannot say whether our results are reflecting a temporary state of the population or more stable differences within it, it is possible that the left tail of our estimate represents a relatively small population of people who differ from the rest in terms of their resilience to adversities and mental health issues. Psychological resilience is associated with comparatively low levels of depression (Imran et al., 2024) and, like depression, seems to be the result of multiple interacting internal and external factors—both somewhat stable inherent tendencies and changing situational factors (Ungar & Theron, 2020). Our results suggest there is a need to study which factors separate resilient or non-depressed people from the others. There has been growing interest in this area of study (e.g., Hoorelbeke et al., 2019; Navrady et al., 2018; Robinson et al., 2014), and our results give tentative support for the increasing focus on resilience and its implications. While it is unethical to experimentally expose the potentially resilient individuals to adversity merely to verify their resilience, statistical counterfactual inference could allow for quantitative estimates on what would have happened had we done so—representing one promising research angle to resilience (Hernán & Robins, 2020; Luque-Fernandez et al., 2018; van der Laan & Rose, 2011).

In line with Falconer’s (1965) and Plomin et al.’s (2009) theories and the findings from psychometric approaches (e.g., Haslam et al., 2020), our results support the continuity of latent depression—at least for the high-risk population. The lack of discontinuity around higher depression risk also aligns with clinical studies comparing MDD and subthreshold depression (STD). They show qualitative similarity in symptoms, impairment, and risk factors (Ayuso-Mateos et al., 2010; Bertha & Balázs, 2013; L.-S. Chen et al., 2000; Lewinsohn et al., 2000; Moore & Brown, 2012). Furthermore, the dose–response connection between the number and frequency of depression symptoms and severity of outcomes is monotone (Lewinsohn et al., 2000; Zuithoff et al., 2010). STD is a well-documented risk factor for MDD (Bertha & Balázs, 2013; Cuijpers & Smit, 2004; Lee et al., 2019; R. Zhang, Peng, et al., 2023) and transitions between these categories are fluid (L.-S. Chen et al., 2000). Interventions are also effective on both STD and MDD (Cuijpers et al., 2014). Taken together, clinical significance of depression symptoms does not seem dependent on whether the patient surpasses the diagnostic threshold or not (Bertha & Balázs, 2013; Lewinsohn et al., 2000), and STD and MDD seem to represent the same latent continuum.

Continuity of latent depression was more ambiguous around the lower end of the risk, as a second mode emerged both in our averaged subsample density and in the BIC-selected model for the total data. Some of the individual estimates for our 12 resamples also had a clear second mode toward the left end of the trait. It is unclear whether these differences between samples reflect true differences in the data or are accounted for by mere noise in sampling and estimation procedures. The simulation estimates from bimodal data give us some insight. We found indications of quantitative inaccuracy, as the height of the first mode was systematically underestimated in the individual estimates. This introduces (non-true) skew to estimated latent distribution that may explain why classic IRT may estimate discrimination parameter more accurately than DC-IRT in some bimodal-data simulations (Supplementary Figure S5)—especially skewness seems to play a role in bias-correction for the discrimination parameter, with normal models performing well under modest skew and failing for stronger skew (Woods, 2007). In addition, 1 of the 10 estimates contained a qualitative inaccuracy of three modes instead of two (Supplementary Figure S2). These errors seemed more likely to happen when a latent mode or modes largely resided below the ordinal thresholds. The bimodal shape was rather pronounced in our simulated latent data and the true latent density in the general population could be more ambiguous regarding possible multimodality, possibly increasing the likelihood of an erroneous mode. Considering these observations, the multiple clear modes in some estimates could be noise, making the less-wiggly averaged density and the BIC-selected density our best estimates—both being bimodal (Figure 3, two last panels).

Skewness and bimodality have also been found in the within-individual frequency distributions of depression symptoms (Hosenfeld et al., 2015) and negative emotions (Haslbeck et al., 2023) over time. Even though these studies take a very different approach than the current one, they may provide an interpretive lens to our results. Their findings fit the idea that emotions and mood are experienced as discrete states separated by tipping points, with sudden phase shifts instead of gradual transitions in between (Scheffer et al., 2024a, 2024b). Transitional states between the non-depressed and the depressed were indeed much less likely, although some individuals did experience a gradual change, indicating heterogeneity between individuals’ dynamical mood systems (Hosenfeld et al., 2015). In the light of these findings, our estimates can be interpreted as averages of thousands of individuals in different mood states, thus showing a degree of bimodality. This explanation is, of course, highly tentative due to the cross-sectional nature of our study and because, in contrast to our findings, the dynamical systems theory would seem to predict a minor mode at the severe end rather than non-severe end of the depression continuum.

Our study had several noteworthy strengths. It used an exceptionally large and representative sample, one of the most widely used depression inventories and state-of-the-art methods for SNP latent-density estimation. The results need to be interpreted in the light of at least following limitations, however. The DC-IRT methodology relies on given two-parameter item response function or on its ordinal-valued extension (graded response model). Albeit a popular choice, other choices could impact latent-density estimates. Furthermore, although the DC-IRT method showed remarkable ability to “see” below the ordinal thresholds set by the PHQ-9 questionnaire, the questionnaire-item characteristics may nevertheless limit the estimation of the lower tail of the latent density. It could be useful to explore items that are highly similar otherwise but evoke higher endorsement rates.

Our results have several important methodological indications for future research. First, when modeling latent depression, it may help to choose an option that does not assume a latent normal distribution, if such an option is available. Second, methods that specifically require a non-Gaussian distribution to function as intended can be used more confidently. One such model is the Linear Non-Gaussian Acyclic Model and its variants—the methods allow for deducing causality from cross-sectional data using higher-order statistical properties of more complicated distributions (Rosenström, Czajkowski, et al., 2023; Rosenström et al., 2012; Shimizu et al., 2006; Wiedermann et al., 2020). Third, more research and development are needed to extend tools that are only available for Gaussian variables to other distributional contexts. Fourth, to avoid bias in person and item parameters when applying IRT to skewed latent constructs, utilizing a method with fewer latent distribution assumptions, such as DC-IRT, seems recommendable [note, fixing variance of the latent trait *within* an estimator is likely to further help when targeting these parameters (cf. X. Zhang et al., 2021)].

To conclude, we created estimates of the latent continuous density of depression in the general population from ordinal symptom questionnaire-item data of different sample sizes. These estimates consistently point to the latent distribution of depression being left-skewed and bimodal. The DC-IRT proved a powerful tool for latent density estimation, although its accuracy with different sample sizes depends on the information criterion that is used for model selection. Although further investigations on the implications of our findings are warranted, our results show that the typical latent-normality assumption does not hold for depression.

## Supplemental Material

sj-docx-1-asm-10.1177_10731911241275327 – Supplemental material for Non-Gaussian Liability Distribution for Depression in the General Population Supplemental material, sj-docx-1-asm-10.1177_10731911241275327 for Non-Gaussian Liability Distribution for Depression in the General Population by Anna Talkkari and Tom H. Rosenström in Assessment
