# Supplementary material for: Non-Gaussian Liability Distribution for Depression in the General Population
Source: Assessment. 2024 Sep 9;32(6):978–91. doi: 10.1177/10731911241275327 (PMC12290230; doi:10.1177/10731911241275327)
Supplement: sj-docx-1-asm-10.1177_10731911241275327 – Supplemental material for Non-Gaussian Liability Distribution for Depression in the General Population [file sj-docx-1-asm-10.1177_10731911241275327.docx]

SUPPLEMENTARY MATERIAL for:

**Non-Gaussian Liability for Depression in the General Population**

Contents

[Supplementary text 2](#_Toc169769152)

[Supplementary Figures 2](#_Toc169769153)

[Supplementary Figure S1 2](#_Toc169769154)

[Supplementary Figure S2 3](#_Toc169769155)

[Supplementary Figure S3 4](#_Toc169769156)

[Supplementary Figure S4 5](#_Toc169769157)

[Supplementary Figure S5 6](#_Toc169769158)

[Supplementary R Scripts 7](#_Toc169769159)

[A function to standardize the DC-IRT density (cf. Zhang et al., 2021) 7](#_Toc169769160)

[Average NHANES item-category endorsement frequencies by survey (frekvenssit.txt) 8](#_Toc169769161)

[The simulation R script (note: additional replications were run for some conditions) 9](#_Toc169769162)

## Supplementary text

In these supplementary materials, we show examples of Davidian curves (Supplementary Figure S1), additional latent-density estimates from models fitted to data from our bimodal simulation condition (Supplementary Figure S2) and integrated squared errors from models estimated for skewed data without using the Hannan-Quinn information criterion (Supplementary Figure S3). Furthermore, we present additional simulation results on estimation accuracy for latent density (Supplementary Figure S4) and person and item parameters (Supplementary Figure S5) when using different modelling solutions, as well as provide the key parts of our main R scripts (computer codes) to ensure reproducibility of our results. The figures were discussed in the main text, but the R-language scripts are intended mainly for the most avid researchers of highly similar topics. Fitting procedures for the [open-access] NHANES data were similar to the here-described procedures for simulated data.

## Supplementary Figures


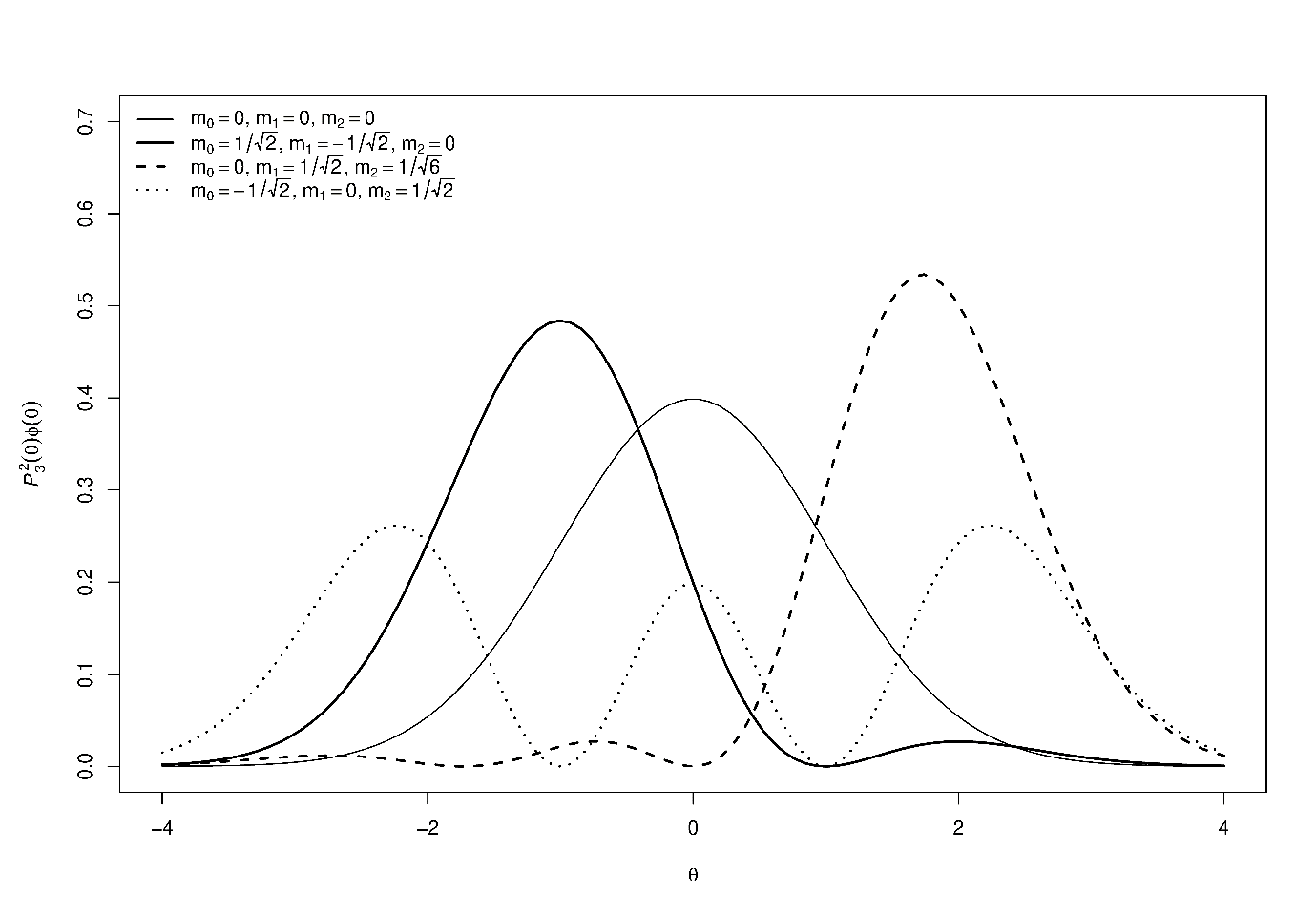


Supplementary Figure S1. *Davidian curves. A few example Davidian curves. Note, all values are not allowed for m_i_. They must satisfy the constraint* $m_{0}^{2}+m_{1}^{2}+3m_{2}^{2}+2m_{0}m_{2}=1$*. Furthermore, in DC-IRT the above densities are sometimes standardized to mean 0 and variance 1. For details, see, e.g., Zhang et al. (2021; from main-text references).*


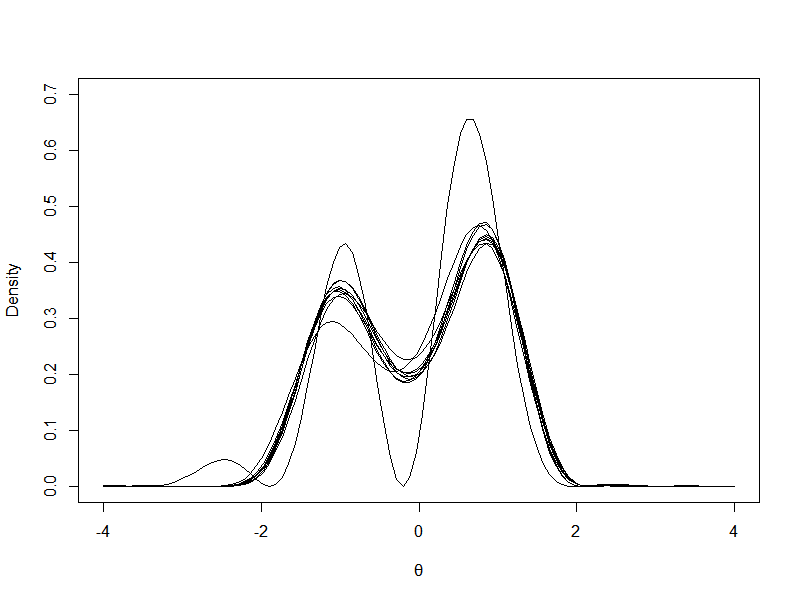


Supplementary Figure S2. *Davidian Curve Item Response Theory estimates of latent density for 10 samples from bimodal simulated data.*





Supplementary Figure S3. *Integrated squared error (ISE) as a function of sample size (n) for our skewed-data simulation condition without (left column) and with (right column) the use of the Hannan-Quinn (HQ) information criterion. “With HQ criterion” refers to setting the polynomial order (i.e., “tuning parameter”) of Davidian-curve model with it, whereas “without HQ criterion” refers to the fixed order 5 (this order was found by HQ criterion for sample size 3000 but could not vary by sample size). In the upper row, the solid line shows the average ISE over 100 (left) or 50 (right) repetitions of each simulation condition, whereas the dotted lines show standard errors of the means and the grey area the 95% confidence interval. The lower row shows the median and inter-quantile range (grey area). Notice how the use of HQ criterion, on average, reduces ISE and its standard error (s.e. is favourable despite using only half the simulations repetitions). However, the average ISE begins to increase again after the sample size grows sufficiently large for the condition with HQ criterion. This behaviour is even more clear from median and inter-quantile range, which reduce the influence of rare outlier estimates. In summary, HQ criterion has poor asymptotic behaviour compared to a fixed model complexity it selected at an intermediate sample size of 3000.*


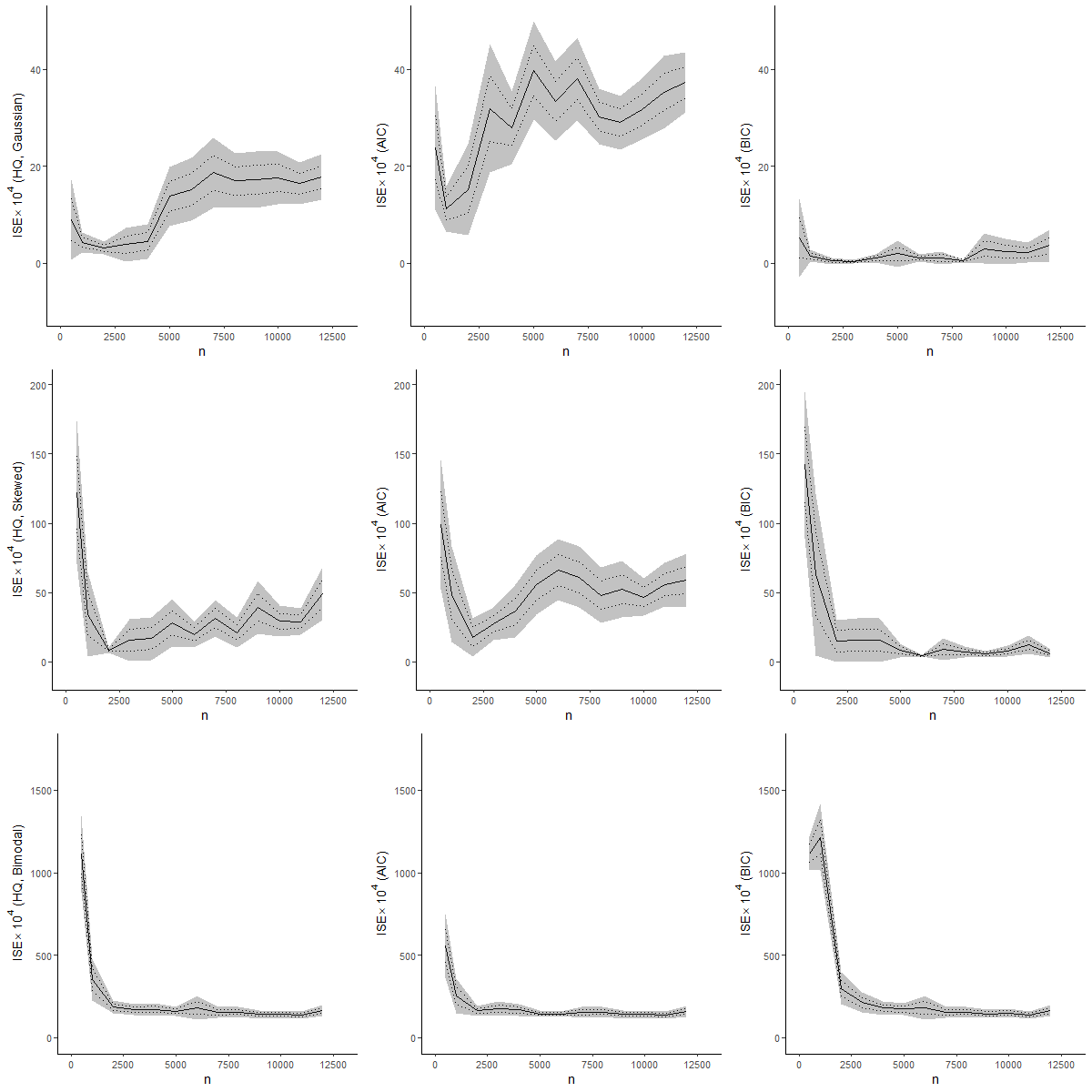


Supplementary Figure S4. *Integrated Squared Error (ISE) as a function of sample size (n) when using Hannan-Quinn (HQ) and Akaike’s (AIC) and Bayesian information criterion (BIC) for the three simulated latent distributions (Gaussian at 1^st^ row, Skewed at 2^nd^, and Bimodal at 3^rd^).*

*
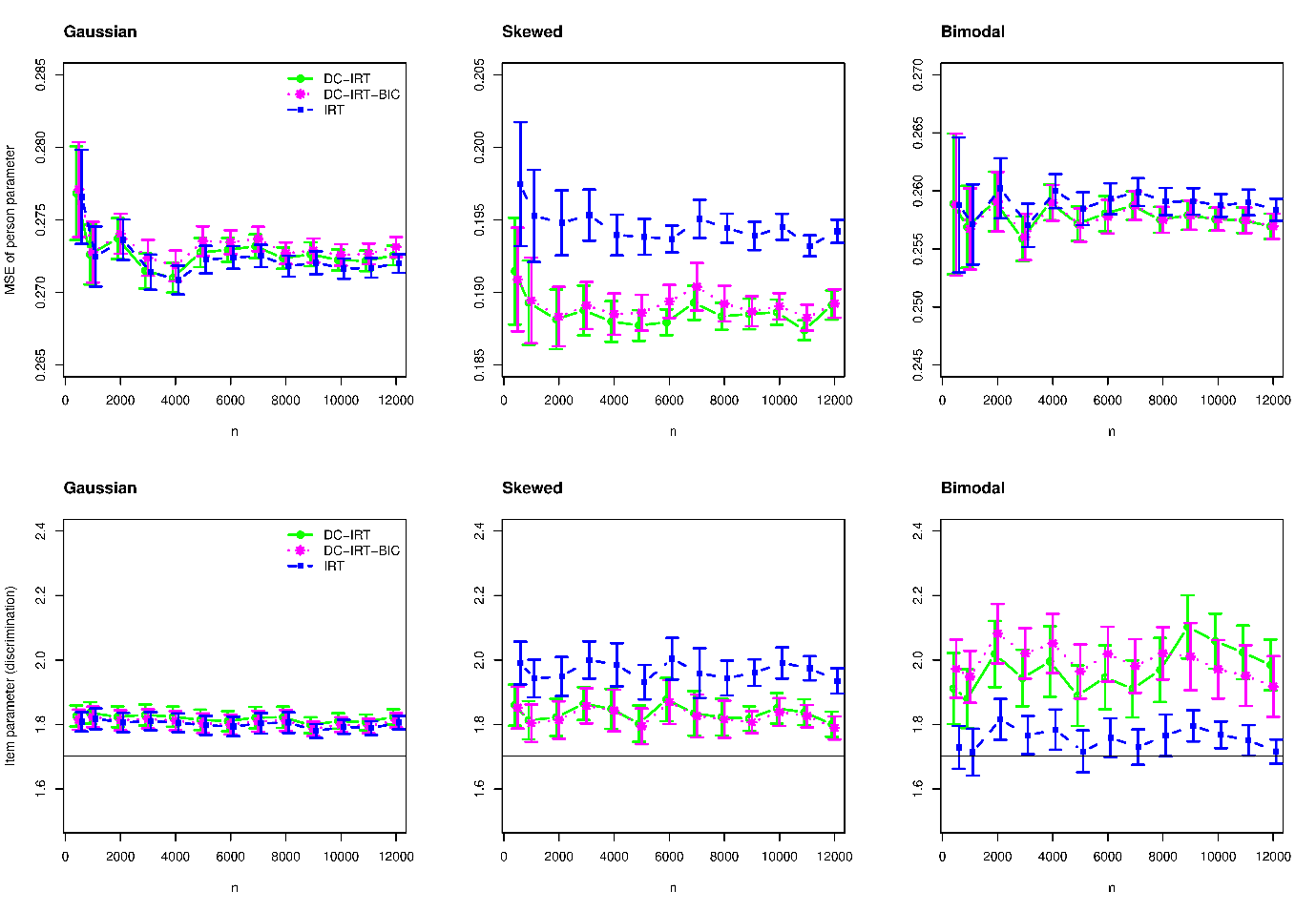
*

Supplementary Figure S5. *Estimation performance for person and item parameters in Davidian Curve Item Response Theory (DC-IRT) compared to traditional IRT. Additionally, Bayesian information criterion (BIC) -based model selection is shown for DC-IRT alongside the recommended HQ-selection. Top row shows mean squared error (MSE) between simulated latent trait and its expected a-posteriori estimate, as given by the default fscores-function of the mirt package. A point estimate of MSE was taken over single simulated sample and the marks correspond to means over the simulation replications and whiskers to their 95% confidence intervals. Bottom row shows the slope (discrimination) estimates given by mirt. The data was simulated with the factor loading √0.5 for each item, which translates to the approximate slope of 1.702 (the mirt correction factor for interpreting Logistic item liabilities as Gaussian ones; note, differs for other factor loadings). All the models were approximations with respect to this parameter, but traditional IRT was the worst approximation in the case of the skewed latent trait, but it may have a very modest advantage for bimodal data.*

## Supplementary R Scripts

### A function to standardize the DC-IRT density (cf. Zhang et al., 2021)

# standardized_DC_density.R

# THR / 2023-09-05

# Implements Eq. 13 from Zhang et al. (2021)

# Multivariate Behavioral Research, 2021;56(5):703-723,

# DOI: 10.1080/00273171.2020.1776096

standardized_DC_density **<-** **function(**x, dcfit**){**

tmp **<-** coef**(**dcfit**)$**GroupPars

k **<-** length**(**tmp**)** **-** 2 # infer k from the DC-IRT model object

Phi **<-** tmp**[**3**:(**k**+**2**)]** # collect Phi parameter vector

cc **<-** sin**(**Phi**[**1**])**

**if** **(**k **>** 1**){**

**for** **(**i **in** 1**:(**k**-**1**)){**cc **<-** c**(**cc, prod**(**cos**(**Phi**[**1**:**i**]))***sin**(**Phi**[**i**+**1**]))}**

cc **<-** c**(**cc, prod**(**cos**(**Phi**[**1**:**k**])))**

**}** **else** **{**

cc **<-** c**(**cc, cos**(**Phi**[**1**]))**

**}**

Mf **<-** **function(**s**){**ss **<-** s**/**2; ifelse**((**s**)**%%2 **==** 0, factorial**(**2*****ss**)/(**2**^**ss*****factorial**(**ss**))**, 0**)}**

M **<-** matrix**(**0,k**+**1,k**+**1**)**; **for** **(**i **in** 1**:(**k**+**1**)){for** **(**j **in** 1**:(**k**+**1**)){** M**[**i,j**]** **<-** i**+**j**-**2**}}**

M **<-** Mf**(**M**)**

B **<-** chol**(**M**)**

m **<-** solve**(**B**)** %*% t**(**t**(**cc**))**

M_star **<-** matrix**(**0,k**+**1,k**+**1**)**; **for** **(**i **in** 1**:(**k**+**1**)){for** **(**j **in** 1**:(**k**+**1**)){** M_star**[**i,j**]** **<-** i**+**j**-**1**}}**

M_star **<-** Mf**(**M_star**)**

M_2stars **<-** matrix**(**0,k**+**1,k**+**1**)**; **for** **(**i **in** 1**:(**k**+**1**)){for** **(**j **in** 1**:(**k**+**1**)){** M_2stars**[**i,j**]** **<-** i**+**j**}}**

M_2stars **<-** Mf**(**M_2stars**)**

mu **<-** c**(**t**(**m**)** %*% M_star %*% m**)**

S **<-** c**(**sqrt**(**t**(**m**)** %*% M_2stars %*% m **-** mu**^**2**))**

fout **<-** rep**(**0, length**(**x**))**

**for** **(**i **in** 1**:**length**(**x**)){**

fout**[**i**]** **<-** sum**(**c**(**m**)** ***** **(**x**[**i**]***S**+**mu**)^(**0**:**k**))^**2 ***** dnorm**(**x**[**i**]***S**+**mu**)** ***** S

**}**

return**(**fout**)**

**}**

### Average NHANES item-category endorsement frequencies by survey (frekvenssit.txt)

The below table of average item-category endorsement frequencies in NHANES surveys is called in the simulation script further below.

Survey Category 0 Category 1 Category 2 Category 3

"05-06" 0.788664211428439 0.148078660273782 0.0342343594376115 0.0290227688601672

"07-08" 0.758738960772232 0.156582460464161 0.0414048059149723 0.0432737728486342

"09-10" 0.758875057630244 0.156253132078497 0.0411129151883256 0.0437588951029326

"11-12" 0.773016517736258 0.145342539940428 0.0390378192977706 0.0426031230255438

"13-14" 0.763255342477089 0.149899666935601 0.0425122571836405 0.0443327334036699

"15-16" 0.76347227632775 0.153767908929576 0.0418993204345756 0.0408604943080985

"17-18" 0.766875068553252 0.147153668970056 0.045124492705934 0.0408467697707579

### The simulation R script (note: additional replications were run for some conditions)

source**(**"standardized_DC_density.R"**)**

### Load packages ###

library**(**mirt**)** # For DC-IRT analyses

library**(**foreach**)** # For parallel computing

library**(**doSNOW**)** # For parallel computing

### Set functions ###

# Skewed distribution with mu = 0, sigma = 1

gs **<-** **function(**x, w**=**0.9, m1**=-**0.2**/**0.9, m2**=**0.2**/**0.1, v1**=**0.450617283950617, v2**=**1.5**){**

return**(** w ***** dnorm**(**x, m1, sqrt**(**v1**))** **+** **(**1**-**w**)** ***** dnorm**(**x, m2, sqrt**(**v2**))** **)**

**}**

gs_sim **<-** **function(**n, w**=**0.9, m1**=-**0.2**/**0.9, m2**=**0.2**/**0.1, v1**=**0.450617283950617, v2**=**1.5**){**

ws **<-** **(**runif**(**n**)** **<=** w**)***1

return**(**ws ***** rnorm**(**n, m1, sqrt**(**v1**))** **+** **(**1**-**ws**)** ***** rnorm**(**n, m2, sqrt**(**v2**)))**

**}**

# Bimodal distribution with mu = o, sigma = 1

gb **<-** **function(**x, w**=**0.5, m1**=-**0.8944, m2**=**0.8944, v1**=**0.2, v2**=**0.20009728**){**

return**(** w ***** dnorm**(**x, m1, sqrt**(**v1**))** **+** **(**1**-**w**)** ***** dnorm**(**x, m2, sqrt**(**v2**))** **)**

**}**

gb_sim **<-** **function(**n, w**=**0.5, m1**=-**0.8944, m2**=**0.8944, v1**=**0.2, v2**=**0.20009728**){**

ws **<-** **(**runif**(**n**)** **<=** w**)***1

return**(**ws ***** rnorm**(**n, m1, sqrt**(**v1**))** **+** **(**1**-**ws**)** ***** rnorm**(**n, m2, sqrt**(**v2**)))**

**}**

### Set parameters ###

latdists **<-** c**(**"Gaussian", "Skewed", "Bimodal"**)**

simdists **<-** list**(**rnorm, gs_sim, gb_sim**)**

dendists **<-** list**(**dnorm, gs, gb**)**

n **<-** c**(**500, 1000, 2000, 3000, 4000, 5000, 6000,

7000, 8000, 9000, 10000, 11000, 12000**)**

# Load average item endorsement frequencies

dfreq **<-** read.table**(**"frekvenssit.txt"**)**

mfreq **<-** colMeans**(**dfreq**[**,2**:**5**])**

# Estimate Gaussian thresholds from the endorsement frequencies

cumth **<-** cumsum**(**mfreq**[**1**:**3**])**

quadists **<-** list**(**qnorm**(**cumth**)**,

c**(**optimise**(function(**x**)** **(**integrate**(**gs, lower **=** **-Inf**, upper **=** x**)$**value **-** cumth**[**1**])^**2,c**(-**3,4**))$**minimum,

optimise**(function(**x**)** **(**integrate**(**gs, lower **=** **-Inf**, upper **=** x**)$**value **-** cumth**[**2**])^**2,c**(-**3,4**))$**minimum,

optimise**(function(**x**)** **(**integrate**(**gs, lower **=** **-Inf**, upper **=** x**)$**value **-** cumth**[**3**])^**2,c**(-**3,4**))$**minimum**)**,

c**(**optimise**(function(**x**)** **(**integrate**(**gb, lower **=** **-Inf**, upper **=** x**)$**value **-** cumth**[**1**])^**2,c**(-**3,4**))$**minimum,

optimise**(function(**x**)** **(**integrate**(**gb, lower **=** **-Inf**, upper **=** x**)$**value **-** cumth**[**2**])^**2,c**(-**3,4**))$**minimum,

optimise**(function(**x**)** **(**integrate**(**gb, lower **=** **-Inf**, upper **=** x**)$**value **-** cumth**[**3**])^**2,c**(-**3,4**))$**minimum**))**

### Define the criterion ###

# A function to evaluate the integrated squared error (ISE)

ISE **<-** **function(**g_hat, g**){**

f **<-** **function(**x**){(**g_hat**(**x**)** **-** g**(**x**))^**2**}**

integrate**(**f, lower **=** **-Inf**, upper **=** **Inf)**

**}**

### Simulate (right-sampling questionnaire) ###

# Activate parallel cluster

# cores <- parallel::detectCores() # Use this for automation

cores **<-** 10

cl **<-** makeCluster**(**cores**)** #cl <- makeCluster(length(n))

nsim **<-** cores*****5

registerDoSNOW**(**cl**)**

t0 **<-** proc.time**()**

allres <- vector("list",3) # Collect ISEs of each condition here

**for** **(**isd **in** 1**:**length(simdists)**){**

# Compute different sample sizes in parallel

lres **<-** foreach**(**isim **=** 1**:**nsim**)** %dopar% **{**

library**(**mirt**)** # needs to be loaded to the parallel clusters too

# Simulate data

res **<-** rep**(**0,length**(**n**))**

**for** **(**ii **in** 1**:**length**(**n**)){**

x **<-** simdists**[[**isd**]](**n**[**ii**])** # latent variable

xx **<-** matrix**(**0,n**[**ii**]**,9**)** # For item liabilities

**for** **(**i **in** 1**:**9**){**xx**[**,i**]** **<-** x**/**sqrt**(**2**)** **+** rnorm**(**n**[**ii**])/**sqrt**(**2**)}**

# Make items that sample high latent-trait values

ds **<-** data.frame**(**xx**)**

names**(**ds**)** **<-** paste0**(**"V",1**:**9**)**

**for** **(**i **in** 1**:**9**){**ds**[**,i**]** **<-** cut**(**ds**[**,i**]**, breaks **=** c**(-Inf**, as.numeric**(**quadists**[[**isd**]])**, **Inf))}**

ds **<-** sapply**(**ds, **function(**x**)** as.numeric**(**ordered**(**x, levels **=** levels**(**ds**[**,1**]))))**

# Fit DC-IRT models to items sampling positive end of the trait

dav **<-** vector**(**"list", 9**)**

**for** **(**i **in** 1**:**9**)** dav**[[**i**]]** **<-** mirt**(**ds, 1, dentype **=** paste0**(**'Davidian-',i**+**1**)**,

technical**=**list**(**NCYCLES **=** 10000**))**

# Compute the HQ criteria

davHQs **<-** rep**(**0,9**)**

**for** **(**i **in** 1**:**9**)** davHQs**[**i**]** **<-** extract.mirt**(**dav**[[**i**]]**, what **=** "HQ"**)**

# Evaluate HQ-best model

dav_HQb **<-** dav**[[**which.min**(**davHQs**)]]**

g_est **<-** **function(**x**)** standardized_DC_density**(**x, dcfit **=** dav_HQb**)**

a **<-** ISE**(**dendists**[[**isd**]]**, g_est**)**

res**[**ii**]** **<-** a**$**value

**}**

res

**}**

allres[[isd]] <- lres

runtime <- proc.time() - t0

**}**

runtime **<-** proc.time**()** **-** t0 # 16.1 h

save**(**allres, runtime, file **=** "simulation_results.Rdata"**)**

stopCluster**(**cl**)**
